# Synthesis and crystal structure of 1,3,5-tris­[(1*H*-benzotriazol-1-yl)meth­yl]-2,4,6-tri­ethyl­benzene

**DOI:** 10.1107/S2056989024009988

**Published:** 2024-10-31

**Authors:** Niklas Koch, Sebastian Förster, Monika Mazik

**Affiliations:** a Institut für Organische Chemie Technische Universität Bergakademie, Freiberg, Leipziger Str 29 09599 Freiberg/Sachsen Germany; Tokyo University of Science, Japan

**Keywords:** crystal structure, tripodal mol­ecule, hydrogen bonding, C—H⋯π inter­actions

## Abstract

In the crystal structure of the title compound, C_33_H_33_N_9_, the tripodal mol­ecule exists in a conformation in which the substituents attached to the central arene ring are arranged in an alternating order above and below the ring plane. The three benzotriazolyl moieties are inclined at angles of 88.3 (1), 85.7 (1) and 82.1 (1)° with respect to the mean plane of the benzene ring. In the crystal, only weak mol­ecular cross-linking involving C—H⋯N hydrogen bonds is observed.

## Abstract 2

The tripodal title mol­ecule exists in a conformation in which the substituents attached to the central arene ring are arranged in an alternating order above and below the ring plane. In the crystal, only weak mol­ecular cross-linking involving C—H⋯N hydrogen bonds is observed.

## 1. Chemical context

Benzotriazole and its derivatives have found applications as auxiliaries in a variety of synthetic strategies (Katritzky & Rachwal, 2010 ▸, 2011 ▸). In addition, numerous benzotriazole derivatives have valuable biological properties, including anti­bacterial, anti­viral, anti­fungal, anti­cancer and others (for reviews, see: Bajaj & Sakhuja, 2015 ▸; Briguglio *et al.*, 2015 ▸). Benzotriazole has also been used as a building block in various supra­molecular architectures. As an example, a water-soluble cavitand bearing a benzotriazole upper rim can be mentioned (Rahman *et al.*, 2022 ▸). Moreover, anti­corrosive low mol­ecular weight gelators based on compounds with benzotriazolyl units have been developed (Cai *et al.*, 2011 ▸). It should also be noted that many benzotriazole-based compounds have been considered in the development of coordination polymers and organometallic frameworks (Loukopoulos & Kostakis, 2019 ▸). These compounds include, for example, benzene derivatives with two 1*H*-benzotriazol-1-ylmethyl groups (Loukopoulos *et al.*, 2018**a* ▸,b* ▸).
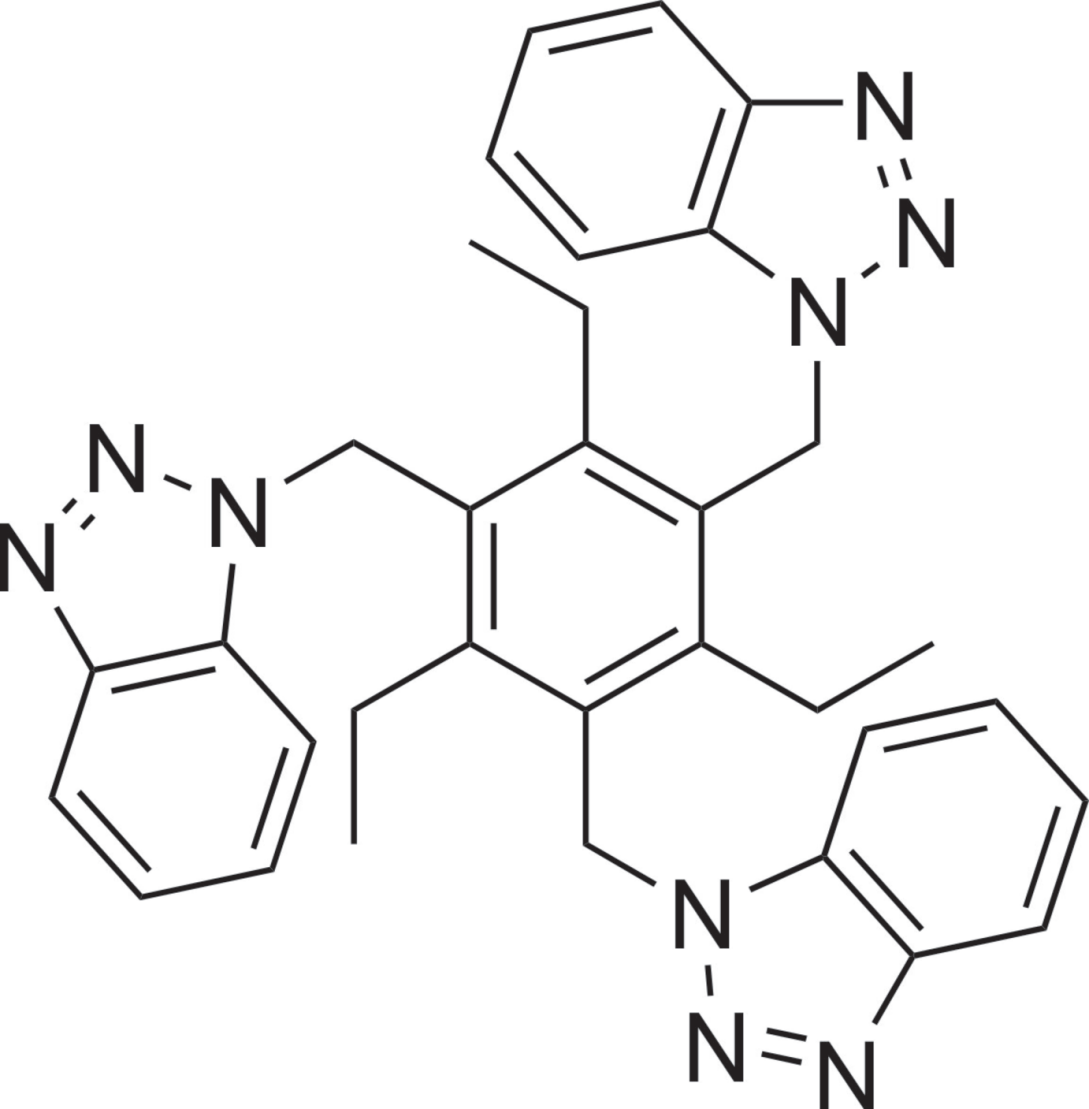


In this article, we describe the synthesis and crystal structure of a compound belonging to the class of 1,3,5-substituted 2,4,6-tri­ethyl­benzenes and bearing 1*H*-benzotriazol-1-ylmethyl substituents. Representatives of this class of compounds have been used by us in the development of artificial receptors for various neutral and ionic substrates, such as carbohydrates (Mazik, 2009 ▸, 2012 ▸; Mazik *et al.*, 2004 ▸, 2005 ▸; Lippe *et al.*, 2015 ▸; Koch *et al.*, 2016 ▸; Kaiser *et al.*, 2019 ▸; Stapf *et al.*, 2020 ▸; Köhler *et al.*, 2020 ▸, 2021 ▸, 2024 ▸), ammonium ions (Schulze *et al.*, 2018 ▸; Fuhrmann *et al.*, 2022*a* ▸,*b* ▸) and hydro­nium/hydroxide ions (Stapf *et al.*, 2015 ▸).

## 2. Structural commentary

**Figure 1:**
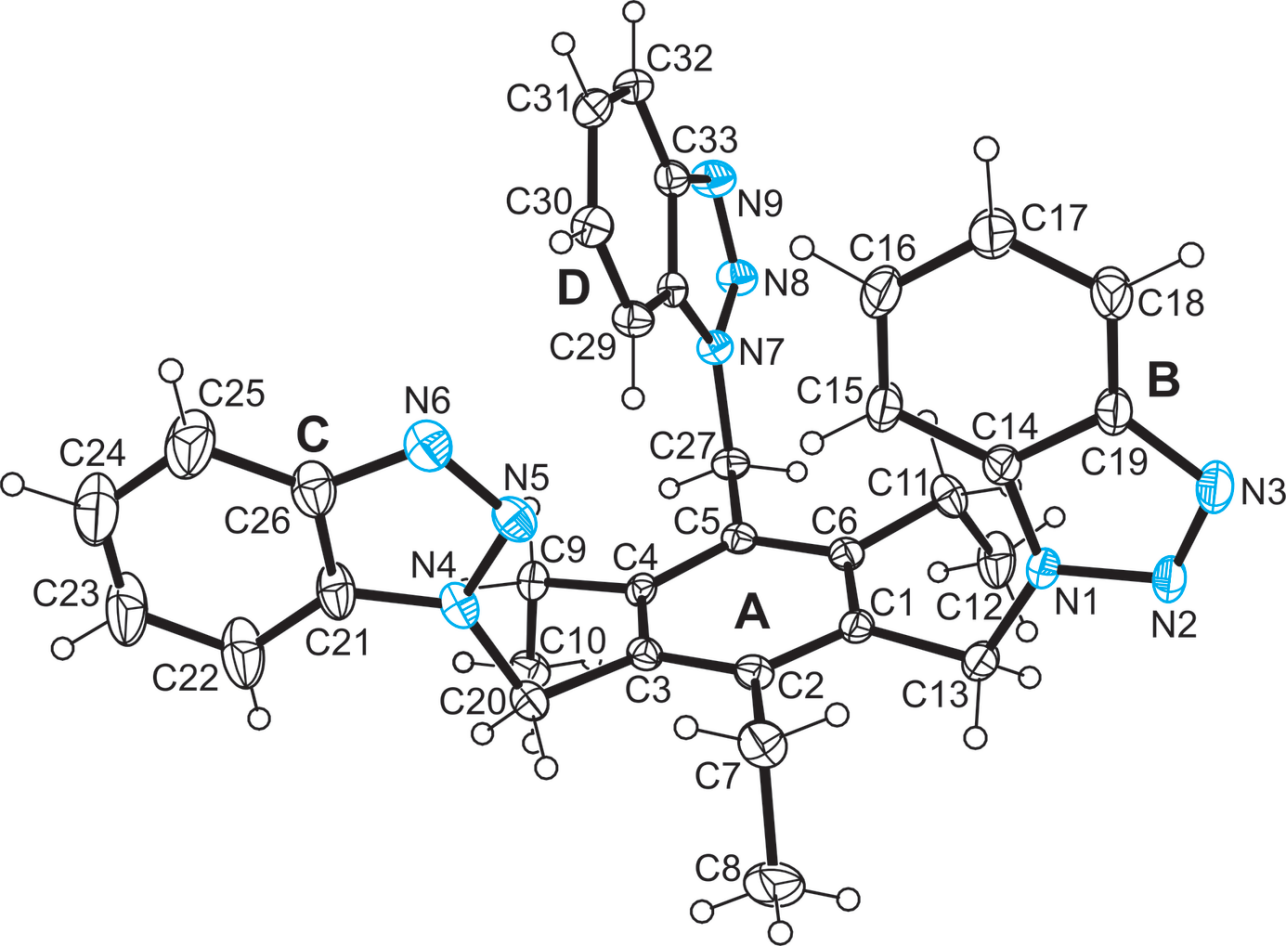
Perspective view of the title mol­ecule including atom labeling and ring specification (A–D). Anisotropic displacement ellipsoids are drawn at the 50% probability level.

The crystal structure of the title compound, C_33_H_33_N_9_, was solved in the ortho­rhom­bic space group *P*2_1_2_1_2_1_ with the asymmetric unit containing one mol­ecule (Fig. 1 ▸). The mol­ecule adopts a conformation in which the benzotriazolyl units are located on one side of the central arene ring, while the ethyl groups are oriented in the opposite direction (*ababab* arrangement, *a* = above, *b* = below; Das & Barbour, 2008*a* ▸,*b* ▸, 2009 ▸; Arunachalam *et al.*, 2010 ▸; Arunachalam & Ghosh, 2010 ▸; Koch *et al.*, 2015 ▸). The dihedral angles between the planes of the benzotriazolyl moieties are 13.6 (1), 88.0 (1) and 76.8 (1)°. The central arene ring of the mol­ecule is noticeably twisted, with the largest atomic distance from the least-squares plane of the ring being 0.048 (1) Å for atom C1 and 0.040 (1) Å for atom C4. The N atoms of two benzotriazolyl moieties (labeled B and D) are directed outwards, while those of the remaining benzotriazolyl unit are directed towards the central arene ring. The distances of 2.76 and 2.96 Å between the arene H atoms H15 and H29 to the center of the benzene ring and the bond geometries (C—H⋯*Cg* = 140°) indicate the presence of two intra­molecular C—H⋯π contacts (Nishio *et al.*, 2009 ▸; Nishio, 2011 ▸; Tiekink & Zukerman-Schpector, 2012 ▸). In addition, an intra­molecular C—H⋯N bond involving the atoms H11*A* and N1 [*d*(H⋯N) 2.54 Å, 133°; Table 1 ▸] is likely to have an influence on the conformation of the mol­ecule.

## 3. Supra­molecular features

**Figure 2:**
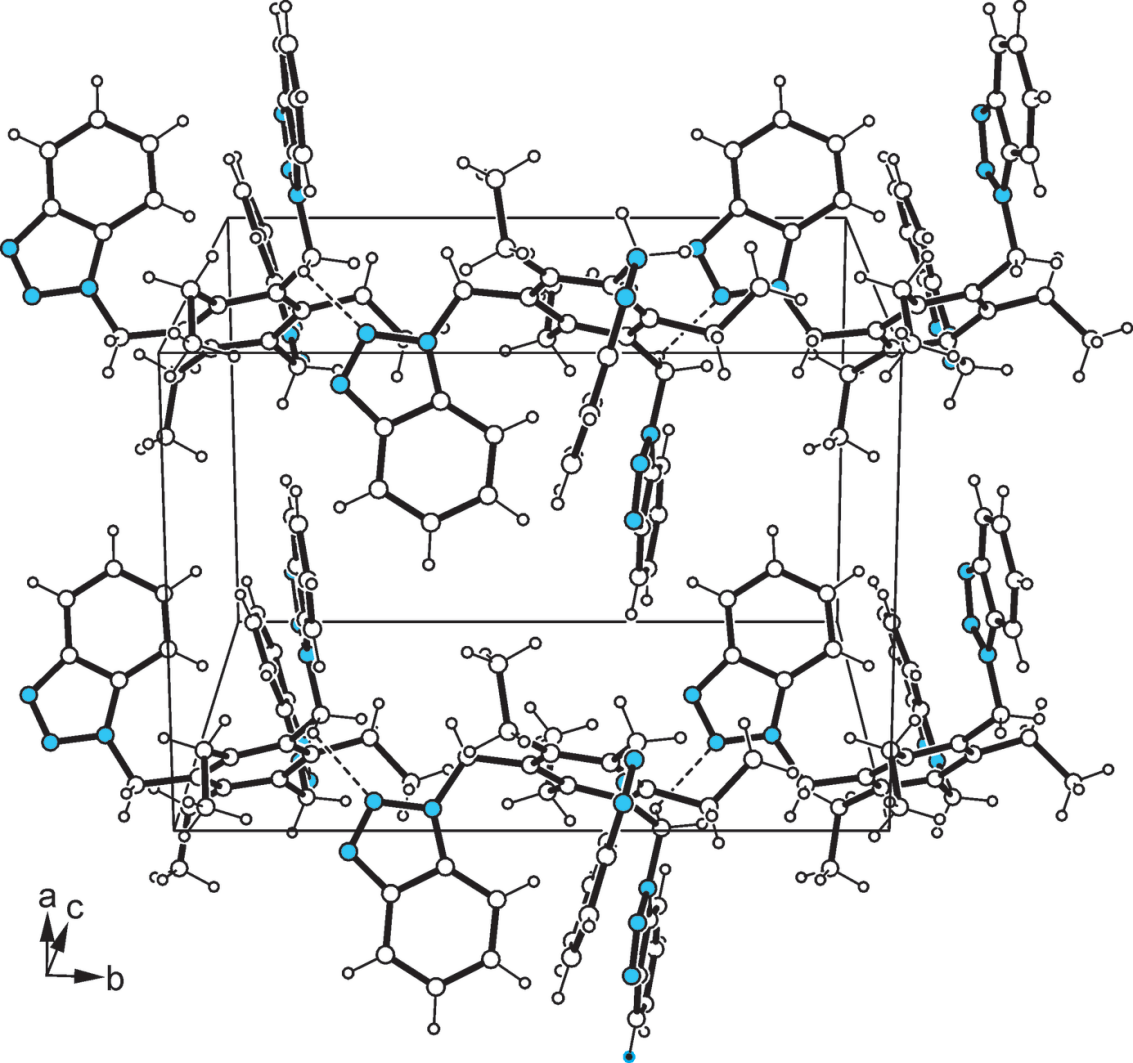
Illustration of the packing structure of the title compound. The dashed lines represent C—H⋯N bonds.

The crystal structure of the title compound is characterized by a low degree of mol­ecular cross-linking. Only the methyl­ene H atoms H20*A* and H27*A* and the N atoms N8 and N3 of adjacent mol­ecules are involved in C—H⋯N hydrogen bonding [*d*(H⋯N) 2.59, 2.64 Å; 140.2° (Table 1 ▸); for other examples of C—H⋯N bonds, see: Desiraju & Steiner, 1999 ▸; Thalladi *et al.*, 2000*a* ▸,*b* ▸; Reddy *et al.*, 1996 ▸; Mazik *et al.*, 1999 ▸, 2000*a* ▸,*b* ▸, 2001 ▸, 2005 ▸]. Consequently, van der Waals forces play an important role in the cohesion of the crystal structure. An excerpt of the packing structure is shown in Fig. 2 ▸.

## 4. Database survey

A search in the Cambridge Structural Database (CSD, Version 5.45, update June 2024; Groom *et al.*, 2016 ▸) for 1,3,5-substituted 2,4,6-tri­alkyl­benzene derivatives bearing three 1*H*-benzotriazol-1-ylmethyl units gave no hits. However, the crystal structures of related tripodal mol­ecules, *e.g.* those equipped with indazolyl (benzopyrazol­yl) moieties, allow a comparison with the crystal structure of the title compound **1**. In the solvent-free crystal structure of 1,3,5-tris­(1*H*-indazolyl-1-yl)-2,4,6-tri­ethyl­benzene (QIDVIL; Schulze *et al.*, 2018 ▸), the mol­ecules adopt a conformation in which two indazolyl units point to the same face of the central benzene ring, while the third points in the opposite direction (*aab* arrangement of the functionalized side arms, *a* = above, *b* = below). By taking the ethyl groups into account, the conformation of the mol­ecule can be defined as *ab′ab′ba′* (position of the ethyl groups are marked as *a′* and *b′*, *a′* = ethyl above, *b′* = ethyl below; Schulze *et al.*, 2017 ▸; Koch *et al.*, 2017 ▸).

For benzene derivatives with 1*H*-benzotriazol-1-ylmethyl groups, only crystal structures of mono- and disubstituted derivatives are known, which, however, often contain further substituents on the benzene ring, such as Br, NO_2_, CN or PhOCH_2_. In addition, the crystal structures of their metal complexes rather than those of the free ligands are mostly reported. As an example of the latter, the crystal structure of 1,3-bis­(1*H*-benzotriazol-1-ylmeth­yl)benzene should be mentioned (AMEZEZ; Macías *et al.*, 2016 ▸). In this case, the benzotriazolyl units form dihedral angles of 88.74 (11) and 85.83 (10)° with the central aromatic ring, which are similar to those observed for two heterocyclic moieties of **1**. The crystal structure is mainly governed by C—H⋯N and C—H⋯π inter­actions.

## 5. Synthesis and crystallization

**Figure 3:**
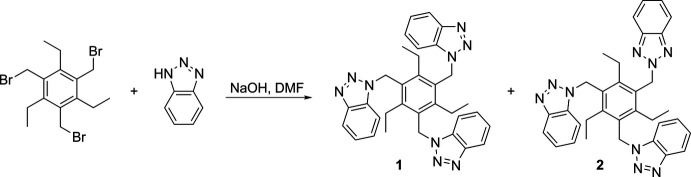
Synthesis of the title compound **1** and the byproduct **2** by reaction of 1*H*-benzotriazole and 1,3,5-tris­(bromo­meth­yl)-2,4,6-tri­ethyl­benzene.

To a suspension of sodium hydroxide (204 mg, 5.10 mmol) in 10 mL of *N,N*-di­methyl­formamide, 1*H*-benzotriazole (608 mg, 5.10 mmol) was added and the mixture was stirred for 20 minutes at room temperature. After addition of 1,3,5-tris­(bromo­meth­yl)-2,4,6-tri­ethyl­benzene (500 mg, 1.13 mmol), the solution was stirred for several hours at room temperature (the course of the reaction was analyzed using TLC). The mixture was then added to 30 mL of ice water, the resulting precipitate was filtered off, washed with small portions of ice water and dried. The crude product was purified by column chromatography [SiO_2_, EtOAc/*n*-hexane *v*/*v* 2:1]. This procedure yielded the compound **1** (340 mg, 0.61 mmol, 54%) and the structure isomer **2** (218 mg, 0.39 mmol, 35%), bearing two 1*H*-benzotriazol-1-ylmethyl units and one 2*H*-benzotriazol-2-ylmethyl group (Fig. 3 ▸). Crystals of the title compound suitable for single crystal X-ray diffraction were grown by slow evaporation of the solvent at room temperature. M.p. 481 K. ^1^H NMR (500 MHz, CDCl_3_, ppm): *δ* = 0.96 (*t*, *J* = 7.5 Hz, 9H, CH_3_), 2.86 (*q*, *J* = 7.5 Hz, 6H, CH_2_), 5.94 (*s*, 6H, CH_2_), 7.03–7.06 (*m*, 3H, H_Ar_), 7.22–7.27 (*m*, 3H, H_Ar_), 7.29–7.33 (*m*, 3H, H_Ar_), 8.00–8.04 (*m*, 3H, H_Ar_).^13^C NMR (125 MHz, DMSO-*d*_6_, ppm) *δ* = 15.1, 23.9, 47.1, 109.9, 120.1, 123.9, 127.7, 129.3, 132.9, 146.3, 146.9. MS (ESI): *m*/*z* calculated for C_33_H_33_N_9_Na [*M* + Na]^+^: 578.2; found 578.2.

## 6. Refinement

Crystal data, data collection and structure refinement details are summarized in Table 2 ▸. The non-hydrogen atoms were refined anisotropically. All hydrogen atoms were positioned geometrically and refined isotropically using the riding model with C—H = 0.98–0.99 Å (alk­yl), 0.95 Å (ar­yl); *U*_iso_(H)= 1.2–1.5*U*_eq_(C).

## Supplementary Material

Crystal structure: contains datablock(s) I. DOI: 10.1107/S2056989024009988/jp2011sup1.cif

Structure factors: contains datablock(s) I. DOI: 10.1107/S2056989024009988/jp2011Isup2.hkl

Supporting information file. DOI: 10.1107/S2056989024009988/jp2011Isup3.cml

CCDC reference: 2391226

Additional supporting information:  crystallographic information; 3D view; checkCIF report

## Figures and Tables

**Table 1:** Hydrogen-bond geometry (Å, °) *Cg*1 represents the centroid of the C1–C6 ring.

*D*—H⋯*A*	*D*—H	H⋯*A*	*D*⋯*A*	*D*—H⋯*A*
C20—H20*A*⋯N8^i^	0.99	2.59	3.341 (3)	133
C27—H27*A*⋯N3^ii^	0.99	2.65	3.217 (3)	117
C11—H11*A*⋯N1	0.99	2.54	3.296 (3)	133
C15—H15⋯*Cg*1	0.95	2.96	3.741 (3)	140
C29—H29⋯*Cg*1	0.95	2.77	3.546 (3)	140

Symmetry codes: (i) 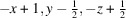; (ii) 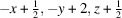.

**Table 2:** Experimental details

Crystal data	
Chemical formula	C_33_H_33_N_9_
*M*_r_	555.68
Crystal system, space group	Orthorhombic, *P*2_1_2_1_2_1_
Temperature (K)	100
*a*, *b*, *c* (Å)	11.0204 (4), 16.1387 (6), 16.2772 (6)
*V* (Å^3^)	2894.98 (18)
*Z*	4
Radiation type	Mo *K*α
μ (mm^−1^)	0.08
Crystal size (mm)	0.42 × 0.18 × 0.17

Data collection	
Diffractometer	Bruker Kappa APEXII CCD area detector
No. of measured, independent and observed [*I* > 2σ(*I*)] reflections	25748, 6002, 5387
*R*_int_	0.033
(sin θ/λ)_max_ (Å^−1^)	0.628

Refinement	
*R*[*F*^2^ > 2σ(*F*^2^)], *wR*(*F*^2^), *S*	0.035, 0.081, 1.05
No. of reflections	6002
No. of parameters	382
H-atom treatment	H-atom parameters constrained
Δρ_max_, Δρ_min_ (e Å^−3^)	0.15, −0.19
Absolute structure	Flack *x* determined using 2115 quotients [(*I*^+^)−(*I*^−^)]/[(*I*^+^)+(*I*^−^)] (Parsons *et al.*, 2013 ▸)

Computer programs: *APEX2* and *SAINT* (Bruker, 2014 ▸), *SHELXS97* (Sheldrick, 2008 ▸), *SHELXL2018/3* (Sheldrick, 2015 ▸), *XP* (Sheldrick, 2008 ▸), *WinGX* (Farrugia, 2012 ▸), *publCIF* (Westrip, 2010 ▸) and *ShelXle* (Hübschle *et al.*, 2011 ▸).

## supplementary crystallographic information

### 1,3,5-Tris[(1H-benzotriazol-1-yl)methyl]-2,4,6-triethylbenzene Crystal data

**Table:**

C_33_H_33_N_9_	*D*_x_ = 1.275 Mg m^−^^3^
*M**_r_* = 555.68	Mo *K*α radiation, λ = 0.71073 Å
Orthorhombic, *P*2_1_2_1_2_1_	Cell parameters from 7118 reflections
*a* = 11.0204 (4) Å	θ = 2.2–27.4°
*b* = 16.1387 (6) Å	µ = 0.08 mm^−^^1^
*c* = 16.2772 (6) Å	*T* = 100 K
*V* = 2894.98 (18) Å^3^	Irregular, colourless
*Z* = 4	0.42 × 0.18 × 0.17 mm
*F*(000) = 1176	

### 1,3,5-Tris[(1H-benzotriazol-1-yl)methyl]-2,4,6-triethylbenzene Data collection

**Table:**

Bruker Kappa APEXII CCD area detector diffractometer	*R*_int_ = 0.033
phi and ω scans	θ_max_ = 26.5°, θ_min_ = 2.5°
25748 measured reflections	*h* = −13→13
6002 independent reflections	*k* = −16→20
5387 reflections with *I* > 2σ(*I*)	*l* = −18→20

### 1,3,5-Tris[(1H-benzotriazol-1-yl)methyl]-2,4,6-triethylbenzene Refinement

**Table:**

Refinement on *F*^2^	Hydrogen site location: inferred from neighbouring sites
Least-squares matrix: full	H-atom parameters constrained
*R*[*F*^2^ > 2σ(*F*^2^)] = 0.035	*w* = 1/[σ^2^(*F*_o_^2^) + (0.0406*P*)^2^ + 0.3656*P*] where *P* = (*F*_o_^2^ + 2*F*_c_^2^)/3
*wR*(*F*^2^) = 0.081	(Δ/σ)_max_ < 0.001
*S* = 1.05	Δρ_max_ = 0.15 e Å^−^^3^
6002 reflections	Δρ_min_ = −0.19 e Å^−^^3^
382 parameters	Absolute structure: Flack *x* determined using 2115 quotients [(*I*^+^)-(*I*^-^)]/[(*I*^+^)+(*I*^-^)] (Parsons *et al.*, 2013)
0 restraints	

### 1,3,5-Tris[(1H-benzotriazol-1-yl)methyl]-2,4,6-triethylbenzene Special details

**Table:**

Geometry. All esds (except the esd in the dihedral angle between two l.s. planes) are estimated using the full covariance matrix. The cell esds are taken into account individually in the estimation of esds in distances, angles and torsion angles; correlations between esds in cell parameters are only used when they are defined by crystal symmetry. An approximate (isotropic) treatment of cell esds is used for estimating esds involving l.s. planes.

### 1,3,5-Tris[(1H-benzotriazol-1-yl)methyl]-2,4,6-triethylbenzene Fractional atomic coordinates and isotropic or equivalent isotropic displacement parameters (Å^2^)

**Table:**

	*x*	*y*	*z*	*U*_iso_*/*U*_eq_	
C1	0.38004 (19)	0.88951 (13)	0.05254 (11)	0.0177 (4)	
C2	0.45997 (19)	0.83741 (12)	0.09532 (12)	0.0182 (4)	
C3	0.51962 (18)	0.86757 (13)	0.16516 (12)	0.0169 (4)	
C4	0.50175 (17)	0.94963 (12)	0.19122 (11)	0.0156 (4)	
C5	0.43143 (19)	1.00303 (12)	0.14297 (12)	0.0165 (4)	
C6	0.37006 (18)	0.97360 (13)	0.07315 (11)	0.0172 (4)	
C7	0.4830 (2)	0.74986 (13)	0.06507 (13)	0.0266 (5)	
H7A	0.568545	0.734934	0.076171	0.032*	
H7B	0.470442	0.747975	0.004872	0.032*	
C8	0.4002 (3)	0.68626 (14)	0.10584 (16)	0.0400 (6)	
H8A	0.413298	0.687060	0.165383	0.060*	
H8B	0.418765	0.630947	0.084406	0.060*	
H8C	0.315353	0.699898	0.093920	0.060*	
C9	0.55736 (19)	0.97958 (13)	0.27096 (12)	0.0192 (4)	
H9A	0.582015	1.038265	0.265053	0.023*	
H9B	0.630853	0.946576	0.283300	0.023*	
C10	0.4670 (2)	0.97151 (14)	0.34195 (12)	0.0234 (5)	
H10A	0.395038	1.005195	0.330317	0.035*	
H10B	0.505048	0.990816	0.392891	0.035*	
H10C	0.443087	0.913352	0.348085	0.035*	
C11	0.2956 (2)	1.03225 (14)	0.02043 (12)	0.0222 (5)	
H11A	0.290222	1.009402	−0.035894	0.027*	
H11B	0.338169	1.086176	0.017049	0.027*	
C12	0.1676 (2)	1.04660 (17)	0.05315 (14)	0.0329 (6)	
H12A	0.121795	0.994575	0.050980	0.049*	
H12B	0.126717	1.088410	0.019366	0.049*	
H12C	0.171915	1.066014	0.110123	0.049*	
C13	0.30133 (19)	0.85410 (14)	−0.01529 (12)	0.0222 (5)	
H13A	0.295301	0.793391	−0.007575	0.027*	
H13B	0.218588	0.877347	−0.009856	0.027*	
C14	0.45504 (19)	0.89480 (13)	−0.12918 (12)	0.0196 (4)	
C15	0.5667 (2)	0.91672 (15)	−0.09411 (13)	0.0312 (5)	
H15	0.580267	0.914007	−0.036529	0.037*	
C16	0.6553 (2)	0.94227 (18)	−0.14752 (14)	0.0353 (6)	
H16	0.731425	0.959233	−0.125868	0.042*	
C17	0.6380 (2)	0.94438 (18)	−0.23308 (14)	0.0360 (6)	
H17	0.702488	0.962073	−0.267630	0.043*	
C18	0.5300 (2)	0.92138 (18)	−0.26732 (14)	0.0369 (6)	
H18	0.518521	0.921543	−0.325168	0.044*	
C19	0.4370 (2)	0.89761 (14)	−0.21403 (13)	0.0244 (5)	
C20	0.60859 (19)	0.81289 (13)	0.21107 (12)	0.0205 (4)	
H20A	0.596595	0.820114	0.270914	0.025*	
H20B	0.592264	0.754142	0.197499	0.025*	
C21	0.8348 (2)	0.82938 (15)	0.23835 (13)	0.0282 (5)	
C22	0.8514 (2)	0.8141 (2)	0.32251 (15)	0.0438 (7)	
H22	0.785438	0.802500	0.358278	0.053*	
C23	0.9692 (3)	0.8170 (3)	0.34969 (17)	0.0646 (10)	
H23	0.985119	0.807849	0.406328	0.078*	
C24	1.0675 (3)	0.8331 (3)	0.29639 (19)	0.0663 (10)	
H24	1.147464	0.833767	0.318119	0.080*	
C25	1.0509 (2)	0.8478 (2)	0.21464 (17)	0.0468 (7)	
H25	1.117336	0.859029	0.179129	0.056*	
C26	0.9309 (2)	0.84539 (15)	0.18542 (14)	0.0294 (5)	
C27	0.42433 (19)	1.09430 (12)	0.16442 (12)	0.0180 (4)	
H27A	0.342861	1.115597	0.149888	0.022*	
H27B	0.435267	1.101133	0.224410	0.022*	
C28	0.62740 (18)	1.12435 (12)	0.08657 (11)	0.0160 (4)	
C29	0.69299 (18)	1.05078 (13)	0.07298 (12)	0.0196 (4)	
H29	0.662367	0.998252	0.089214	0.023*	
C30	0.80371 (19)	1.05899 (14)	0.03498 (13)	0.0221 (5)	
H30	0.850655	1.010634	0.025116	0.027*	
C31	0.85043 (19)	1.13649 (14)	0.01001 (12)	0.0222 (4)	
H31	0.927340	1.139038	−0.016233	0.027*	
C32	0.7865 (2)	1.20794 (14)	0.02310 (13)	0.0224 (5)	
H32	0.817485	1.260185	0.006310	0.027*	
C33	0.67341 (19)	1.20129 (12)	0.06221 (12)	0.0189 (4)	
N1	0.34537 (15)	0.87056 (10)	−0.09864 (10)	0.0176 (4)	
N2	0.26511 (16)	0.86005 (12)	−0.16098 (11)	0.0235 (4)	
N3	0.31885 (17)	0.87568 (14)	−0.23032 (11)	0.0307 (5)	
N4	0.73447 (16)	0.83275 (11)	0.19011 (10)	0.0204 (4)	
N5	0.76712 (17)	0.84885 (11)	0.11110 (10)	0.0225 (4)	
N6	0.88511 (17)	0.85727 (11)	0.10747 (11)	0.0263 (4)	
N7	0.51729 (15)	1.14327 (10)	0.12093 (10)	0.0172 (3)	
N8	0.49855 (17)	1.22634 (10)	0.11714 (11)	0.0212 (4)	
N9	0.59115 (17)	1.26176 (11)	0.08186 (11)	0.0252 (4)	

### 1,3,5-Tris[(1H-benzotriazol-1-yl)methyl]-2,4,6-triethylbenzene Atomic displacement parameters (Å^2^)

**Table:**

	*U*^11^	*U*^22^	*U*^33^	*U*^12^	*U*^13^	*U*^23^
C1	0.0162 (10)	0.0218 (11)	0.0150 (9)	−0.0036 (8)	0.0016 (8)	0.0000 (8)
C2	0.0208 (11)	0.0169 (10)	0.0169 (9)	−0.0013 (8)	0.0036 (8)	0.0006 (8)
C3	0.0162 (10)	0.0181 (10)	0.0166 (9)	0.0004 (8)	0.0029 (8)	0.0026 (8)
C4	0.0114 (10)	0.0199 (10)	0.0155 (9)	−0.0014 (8)	0.0024 (8)	0.0002 (8)
C5	0.0137 (10)	0.0169 (10)	0.0190 (9)	0.0010 (8)	0.0051 (8)	0.0012 (7)
C6	0.0140 (10)	0.0216 (10)	0.0159 (9)	0.0002 (8)	0.0045 (8)	0.0027 (8)
C7	0.0359 (13)	0.0191 (11)	0.0247 (10)	0.0028 (9)	−0.0025 (10)	−0.0037 (9)
C8	0.0561 (18)	0.0189 (12)	0.0450 (14)	−0.0048 (11)	−0.0052 (13)	0.0033 (11)
C9	0.0185 (11)	0.0204 (11)	0.0187 (9)	0.0014 (9)	−0.0027 (8)	−0.0010 (8)
C10	0.0292 (13)	0.0230 (11)	0.0179 (9)	0.0006 (9)	0.0007 (9)	0.0001 (9)
C11	0.0227 (11)	0.0250 (11)	0.0190 (10)	0.0069 (9)	−0.0016 (9)	0.0008 (9)
C12	0.0222 (12)	0.0463 (15)	0.0301 (12)	0.0116 (11)	−0.0034 (10)	−0.0042 (11)
C13	0.0194 (11)	0.0269 (12)	0.0202 (10)	−0.0054 (9)	−0.0012 (8)	−0.0001 (9)
C14	0.0184 (11)	0.0208 (10)	0.0197 (9)	−0.0006 (8)	−0.0002 (8)	−0.0004 (8)
C15	0.0226 (12)	0.0513 (15)	0.0197 (10)	−0.0093 (11)	−0.0051 (9)	0.0026 (10)
C16	0.0201 (12)	0.0578 (17)	0.0281 (12)	−0.0109 (12)	−0.0051 (10)	0.0031 (12)
C17	0.0225 (12)	0.0604 (17)	0.0251 (11)	−0.0090 (12)	0.0013 (10)	0.0075 (11)
C18	0.0250 (13)	0.0663 (18)	0.0193 (11)	−0.0098 (12)	−0.0018 (10)	0.0076 (11)
C19	0.0191 (11)	0.0335 (13)	0.0206 (10)	−0.0011 (10)	−0.0034 (9)	0.0012 (9)
C20	0.0217 (11)	0.0216 (11)	0.0182 (9)	0.0027 (9)	0.0015 (9)	0.0023 (8)
C21	0.0221 (12)	0.0379 (13)	0.0247 (11)	0.0111 (10)	−0.0027 (9)	−0.0013 (10)
C22	0.0322 (15)	0.077 (2)	0.0224 (11)	0.0135 (14)	−0.0011 (11)	0.0038 (12)
C23	0.0370 (17)	0.130 (3)	0.0266 (13)	0.0156 (19)	−0.0108 (13)	0.0061 (17)
C24	0.0285 (16)	0.123 (3)	0.0472 (17)	0.0109 (19)	−0.0144 (14)	0.0026 (18)
C25	0.0229 (13)	0.074 (2)	0.0432 (15)	0.0060 (14)	0.0010 (12)	0.0021 (14)
C26	0.0248 (12)	0.0363 (13)	0.0270 (11)	0.0088 (10)	0.0009 (10)	−0.0006 (10)
C27	0.0163 (10)	0.0177 (10)	0.0202 (9)	0.0003 (8)	0.0038 (8)	0.0001 (8)
C28	0.0157 (10)	0.0187 (10)	0.0135 (8)	0.0008 (8)	−0.0022 (8)	−0.0008 (8)
C29	0.0209 (11)	0.0154 (10)	0.0224 (10)	0.0018 (8)	0.0024 (8)	0.0007 (8)
C30	0.0207 (11)	0.0219 (11)	0.0238 (10)	0.0042 (9)	0.0012 (9)	−0.0008 (9)
C31	0.0158 (10)	0.0278 (11)	0.0230 (10)	0.0004 (9)	0.0011 (8)	0.0021 (9)
C32	0.0217 (12)	0.0205 (11)	0.0251 (10)	−0.0045 (8)	0.0000 (9)	0.0027 (9)
C33	0.0213 (11)	0.0162 (10)	0.0191 (9)	0.0012 (8)	−0.0033 (9)	0.0000 (8)
N1	0.0168 (9)	0.0184 (8)	0.0176 (8)	−0.0012 (7)	−0.0031 (7)	−0.0015 (7)
N2	0.0208 (9)	0.0299 (10)	0.0199 (8)	−0.0023 (8)	−0.0058 (7)	−0.0010 (8)
N3	0.0219 (10)	0.0516 (13)	0.0186 (8)	−0.0084 (9)	−0.0037 (8)	0.0019 (9)
N4	0.0223 (9)	0.0232 (10)	0.0157 (8)	0.0067 (7)	0.0008 (7)	−0.0003 (7)
N5	0.0262 (10)	0.0228 (9)	0.0183 (8)	0.0049 (8)	0.0017 (7)	0.0014 (7)
N6	0.0239 (10)	0.0282 (10)	0.0267 (9)	0.0065 (8)	0.0039 (8)	−0.0003 (8)
N7	0.0177 (8)	0.0135 (8)	0.0204 (8)	0.0015 (7)	0.0008 (7)	−0.0010 (7)
N8	0.0229 (10)	0.0144 (8)	0.0264 (9)	0.0035 (7)	0.0023 (8)	0.0010 (7)
N9	0.0248 (10)	0.0176 (9)	0.0333 (10)	0.0011 (7)	0.0036 (8)	0.0020 (8)

### 1,3,5-Tris[(1H-benzotriazol-1-yl)methyl]-2,4,6-triethylbenzene Geometric parameters (Å, º)

**Table:**

C1—C6	1.402 (3)	C17—H17	0.9500
C1—C2	1.403 (3)	C18—C19	1.396 (3)
C1—C13	1.516 (3)	C18—H18	0.9500
C2—C3	1.401 (3)	C19—N3	1.375 (3)
C2—C7	1.517 (3)	C20—N4	1.464 (3)
C3—C4	1.404 (3)	C20—H20A	0.9900
C3—C20	1.516 (3)	C20—H20B	0.9900
C4—C5	1.400 (3)	C21—N4	1.358 (3)
C4—C9	1.515 (3)	C21—C26	1.389 (3)
C5—C6	1.405 (3)	C21—C22	1.404 (3)
C5—C27	1.516 (3)	C22—C23	1.372 (4)
C6—C11	1.518 (3)	C22—H22	0.9500
C7—C8	1.525 (3)	C23—C24	1.412 (4)
C7—H7A	0.9900	C23—H23	0.9500
C7—H7B	0.9900	C24—C25	1.364 (4)
C8—H8A	0.9800	C24—H24	0.9500
C8—H8B	0.9800	C25—C26	1.406 (4)
C8—H8C	0.9800	C25—H25	0.9500
C9—C10	1.531 (3)	C26—N6	1.379 (3)
C9—H9A	0.9900	C27—N7	1.475 (3)
C9—H9B	0.9900	C27—H27A	0.9900
C10—H10A	0.9800	C27—H27B	0.9900
C10—H10B	0.9800	C28—N7	1.371 (3)
C10—H10C	0.9800	C28—C33	1.399 (3)
C11—C12	1.526 (3)	C28—C29	1.408 (3)
C11—H11A	0.9900	C29—C30	1.374 (3)
C11—H11B	0.9900	C29—H29	0.9500
C12—H12A	0.9800	C30—C31	1.412 (3)
C12—H12B	0.9800	C30—H30	0.9500
C12—H12C	0.9800	C31—C32	1.368 (3)
C13—N1	1.465 (3)	C31—H31	0.9500
C13—H13A	0.9900	C32—C33	1.403 (3)
C13—H13B	0.9900	C32—H32	0.9500
C14—N1	1.364 (3)	C33—N9	1.370 (3)
C14—C19	1.396 (3)	N1—N2	1.357 (2)
C14—C15	1.402 (3)	N2—N3	1.299 (3)
C15—C16	1.371 (3)	N4—N5	1.360 (2)
C15—H15	0.9500	N5—N6	1.309 (3)
C16—C17	1.406 (3)	N7—N8	1.358 (2)
C16—H16	0.9500	N8—N9	1.303 (2)
C17—C18	1.366 (3)		
			
C6—C1—C2	120.70 (18)	C16—C17—H17	119.5
C6—C1—C13	119.62 (18)	C17—C18—C19	117.5 (2)
C2—C1—C13	119.66 (18)	C17—C18—H18	121.3
C3—C2—C1	119.30 (18)	C19—C18—H18	121.3
C3—C2—C7	120.57 (18)	N3—C19—C14	108.51 (19)
C1—C2—C7	120.13 (18)	N3—C19—C18	130.2 (2)
C2—C3—C4	120.47 (18)	C14—C19—C18	121.2 (2)
C2—C3—C20	120.08 (18)	N4—C20—C3	111.75 (16)
C4—C3—C20	119.39 (17)	N4—C20—H20A	109.3
C5—C4—C3	119.26 (17)	C3—C20—H20A	109.3
C5—C4—C9	120.54 (18)	N4—C20—H20B	109.3
C3—C4—C9	120.20 (17)	C3—C20—H20B	109.3
C4—C5—C6	120.80 (18)	H20A—C20—H20B	107.9
C4—C5—C27	119.84 (18)	N4—C21—C26	104.78 (18)
C6—C5—C27	119.33 (18)	N4—C21—C22	132.6 (2)
C1—C6—C5	118.87 (18)	C26—C21—C22	122.6 (2)
C1—C6—C11	120.70 (18)	C23—C22—C21	115.6 (3)
C5—C6—C11	120.42 (18)	C23—C22—H22	122.2
C2—C7—C8	112.68 (19)	C21—C22—H22	122.2
C2—C7—H7A	109.1	C22—C23—C24	122.3 (3)
C8—C7—H7A	109.1	C22—C23—H23	118.9
C2—C7—H7B	109.1	C24—C23—H23	118.9
C8—C7—H7B	109.1	C25—C24—C23	121.9 (3)
H7A—C7—H7B	107.8	C25—C24—H24	119.0
C7—C8—H8A	109.5	C23—C24—H24	119.0
C7—C8—H8B	109.5	C24—C25—C26	116.8 (3)
H8A—C8—H8B	109.5	C24—C25—H25	121.6
C7—C8—H8C	109.5	C26—C25—H25	121.6
H8A—C8—H8C	109.5	N6—C26—C21	108.5 (2)
H8B—C8—H8C	109.5	N6—C26—C25	130.7 (2)
C4—C9—C10	110.89 (17)	C21—C26—C25	120.8 (2)
C4—C9—H9A	109.5	N7—C27—C5	111.98 (16)
C10—C9—H9A	109.5	N7—C27—H27A	109.2
C4—C9—H9B	109.5	C5—C27—H27A	109.2
C10—C9—H9B	109.5	N7—C27—H27B	109.2
H9A—C9—H9B	108.0	C5—C27—H27B	109.2
C9—C10—H10A	109.5	H27A—C27—H27B	107.9
C9—C10—H10B	109.5	N7—C28—C33	103.82 (17)
H10A—C10—H10B	109.5	N7—C28—C29	134.97 (19)
C9—C10—H10C	109.5	C33—C28—C29	121.20 (18)
H10A—C10—H10C	109.5	C30—C29—C28	116.45 (19)
H10B—C10—H10C	109.5	C30—C29—H29	121.8
C6—C11—C12	113.39 (18)	C28—C29—H29	121.8
C6—C11—H11A	108.9	C29—C30—C31	122.6 (2)
C12—C11—H11A	108.9	C29—C30—H30	118.7
C6—C11—H11B	108.9	C31—C30—H30	118.7
C12—C11—H11B	108.9	C32—C31—C30	120.91 (19)
H11A—C11—H11B	107.7	C32—C31—H31	119.5
C11—C12—H12A	109.5	C30—C31—H31	119.5
C11—C12—H12B	109.5	C31—C32—C33	117.62 (19)
H12A—C12—H12B	109.5	C31—C32—H32	121.2
C11—C12—H12C	109.5	C33—C32—H32	121.2
H12A—C12—H12C	109.5	N9—C33—C28	109.05 (18)
H12B—C12—H12C	109.5	N9—C33—C32	129.72 (19)
N1—C13—C1	114.61 (17)	C28—C33—C32	121.23 (19)
N1—C13—H13A	108.6	N2—N1—C14	109.94 (16)
C1—C13—H13A	108.6	N2—N1—C13	116.99 (16)
N1—C13—H13B	108.6	C14—N1—C13	133.07 (17)
C1—C13—H13B	108.6	N3—N2—N1	109.16 (16)
H13A—C13—H13B	107.6	N2—N3—C19	108.30 (17)
N1—C14—C19	104.09 (18)	C21—N4—N5	109.81 (18)
N1—C14—C15	134.57 (19)	C21—N4—C20	128.95 (17)
C19—C14—C15	121.3 (2)	N5—N4—C20	120.83 (16)
C16—C15—C14	116.29 (19)	N6—N5—N4	108.99 (17)
C16—C15—H15	121.9	N5—N6—C26	107.93 (18)
C14—C15—H15	121.9	N8—N7—C28	109.64 (16)
C15—C16—C17	122.6 (2)	N8—N7—C27	116.44 (16)
C15—C16—H16	118.7	C28—N7—C27	133.74 (16)
C17—C16—H16	118.7	N9—N8—N7	109.52 (16)
C18—C17—C16	121.0 (2)	N8—N9—C33	107.97 (16)
C18—C17—H17	119.5		
			
C6—C1—C2—C3	7.4 (3)	C22—C21—C26—N6	179.7 (2)
C13—C1—C2—C3	−171.07 (17)	N4—C21—C26—C25	179.3 (2)
C6—C1—C2—C7	−171.50 (19)	C22—C21—C26—C25	−0.6 (4)
C13—C1—C2—C7	10.0 (3)	C24—C25—C26—N6	−179.9 (3)
C1—C2—C3—C4	−1.4 (3)	C24—C25—C26—C21	0.4 (4)
C7—C2—C3—C4	177.55 (18)	C4—C5—C27—N7	92.0 (2)
C1—C2—C3—C20	−178.56 (18)	C6—C5—C27—N7	−86.0 (2)
C7—C2—C3—C20	0.3 (3)	N7—C28—C29—C30	179.7 (2)
C2—C3—C4—C5	−5.1 (3)	C33—C28—C29—C30	0.1 (3)
C20—C3—C4—C5	172.09 (17)	C28—C29—C30—C31	−0.4 (3)
C2—C3—C4—C9	174.44 (18)	C29—C30—C31—C32	0.2 (3)
C20—C3—C4—C9	−8.3 (3)	C30—C31—C32—C33	0.1 (3)
C3—C4—C5—C6	5.7 (3)	N7—C28—C33—N9	−0.4 (2)
C9—C4—C5—C6	−173.84 (18)	C29—C28—C33—N9	179.32 (18)
C3—C4—C5—C27	−172.29 (18)	N7—C28—C33—C32	−179.47 (18)
C9—C4—C5—C27	8.1 (3)	C29—C28—C33—C32	0.2 (3)
C2—C1—C6—C5	−6.8 (3)	C31—C32—C33—N9	−179.2 (2)
C13—C1—C6—C5	171.67 (17)	C31—C32—C33—C28	−0.3 (3)
C2—C1—C6—C11	172.39 (18)	C19—C14—N1—N2	−0.6 (2)
C13—C1—C6—C11	−9.1 (3)	C15—C14—N1—N2	177.4 (2)
C4—C5—C6—C1	0.2 (3)	C19—C14—N1—C13	178.9 (2)
C27—C5—C6—C1	178.21 (18)	C15—C14—N1—C13	−3.1 (4)
C4—C5—C6—C11	−179.02 (18)	C1—C13—N1—N2	−163.28 (18)
C27—C5—C6—C11	−1.0 (3)	C1—C13—N1—C14	17.3 (3)
C3—C2—C7—C8	87.5 (3)	C14—N1—N2—N3	0.6 (2)
C1—C2—C7—C8	−93.6 (2)	C13—N1—N2—N3	−178.91 (18)
C5—C4—C9—C10	85.3 (2)	N1—N2—N3—C19	−0.4 (3)
C3—C4—C9—C10	−94.2 (2)	C14—C19—N3—N2	0.1 (3)
C1—C6—C11—C12	97.3 (2)	C18—C19—N3—N2	−178.6 (3)
C5—C6—C11—C12	−83.5 (2)	C26—C21—N4—N5	0.8 (2)
C6—C1—C13—N1	80.3 (2)	C22—C21—N4—N5	−179.2 (3)
C2—C1—C13—N1	−101.3 (2)	C26—C21—N4—C20	173.4 (2)
N1—C14—C15—C16	−176.6 (2)	C22—C21—N4—C20	−6.6 (4)
C19—C14—C15—C16	1.2 (4)	C3—C20—N4—C21	146.5 (2)
C14—C15—C16—C17	−1.9 (4)	C3—C20—N4—N5	−41.7 (2)
C15—C16—C17—C18	0.6 (5)	C21—N4—N5—N6	−1.0 (2)
C16—C17—C18—C19	1.4 (4)	C20—N4—N5—N6	−174.31 (17)
N1—C14—C19—N3	0.3 (3)	N4—N5—N6—C26	0.8 (2)
C15—C14—C19—N3	−178.0 (2)	C21—C26—N6—N5	−0.3 (3)
N1—C14—C19—C18	179.2 (2)	C25—C26—N6—N5	−179.9 (3)
C15—C14—C19—C18	0.8 (4)	C33—C28—N7—N8	0.1 (2)
C17—C18—C19—N3	176.5 (3)	C29—C28—N7—N8	−179.5 (2)
C17—C18—C19—C14	−2.1 (4)	C33—C28—N7—C27	−174.7 (2)
C2—C3—C20—N4	100.5 (2)	C29—C28—N7—C27	5.7 (4)
C4—C3—C20—N4	−76.8 (2)	C5—C27—N7—N8	162.10 (17)
N4—C21—C22—C23	−179.1 (3)	C5—C27—N7—C28	−23.4 (3)
C26—C21—C22—C23	0.8 (4)	C28—N7—N8—N9	0.1 (2)
C21—C22—C23—C24	−0.9 (5)	C27—N7—N8—N9	175.93 (17)
C22—C23—C24—C25	0.8 (6)	N7—N8—N9—C33	−0.3 (2)
C23—C24—C25—C26	−0.5 (5)	C28—C33—N9—N8	0.4 (2)
N4—C21—C26—N6	−0.4 (3)	C32—C33—N9—N8	179.5 (2)

### 1,3,5-Tris[(1H-benzotriazol-1-yl)methyl]-2,4,6-triethylbenzene Hydrogen-bond geometry (Å, º)

*Cg*1 represents the centroid of the C1–C6 ring.

**Table:**

*D*—H···*A*	*D*—H	H···*A*	*D*···*A*	*D*—H···*A*
C20—H20*A*···N8^i^	0.99	2.59	3.341 (3)	133
C27—H27*A*···N3^ii^	0.99	2.65	3.217 (3)	117
C11—H11*A*···N1	0.99	2.54	3.296 (3)	133
C15—H15···*Cg*1	0.95	2.96	3.741 (3)	140
C29—H29···*Cg*1	0.95	2.77	3.546 (3)	140

Symmetry codes: (i) −*x*+1, *y*−1/2, −*z*+1/2; (ii) −*x*+1/2, −*y*+2, *z*+1/2.
